# Global Health Challenges: The Need for an Expanded Discourse on Bioethics

**DOI:** 10.1371/journal.pmed.0020143

**Published:** 2005-07-26

**Authors:** Solomon R Benatar, Abdallah S Daar, Peter A Singer

## Abstract

Benatar and colleagues argue that the world has changed profoundly since the birth of modern bioethics in the 1960s, and that bioethics needs to address today's global health problems.

Although the 20th century saw a major expansion of the world economy, impressive military/security advances, and spectacular progress in science and technology, the grim reality in the first decade of the new millennium is that human life, health, and security remain under severe threat—but now from the adverse effects of inexorably widening disparities in wealth, health, and knowledge within and between nations. The gap between the income of the richest and poorest 20% of people in the world increased from a 9-fold difference at the beginning of the 20th century to 30-fold by 1960—and since then to over 80-fold by 2000 (Figure 1). Although life expectancy has improved dramatically worldwide during this century, this trend has been reversed in the poorest countries in recent years [1]. The challenge of achieving improved health for a greater proportion of the world's population is one of the most pressing problems of our time and is starkly illustrated by the threat of infectious diseases.

**Figure 1:**
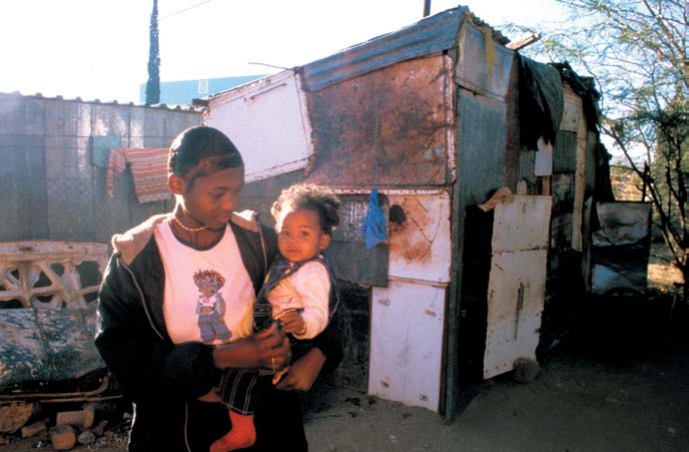
Namibia's Worst Slum—Katutura in Windhoek The gap between the income of the richest and poorest 20% of people in the world increased from a 9-fold difference at the beginning of the 20th century to over 80-fold by 2000. (Photo: Jacob Holdt, www.american-pictures.com)

## Values

The underlying basis for new threats to health, life, and security is our failure to adequately pursue the values that play an essential role in improving population health locally and globally. Such values include meaningful respect for human life, human rights, equity, freedom, democracy, environmental sustainability, and solidarity. Foremost among these is solidarity—without it, we ignore distant indignities, violations of human rights, inequities, deprivation of freedom, undemocratic regimes, and damage to the environment.

We argue that this set of values—which combines genuine respect for the dignity of all people with a desire to promote the idea of human development beyond that conceived within the narrow, individualistic “economic” model of human flourishing—could serve to promote peaceful and beneficial use of new knowledge and power [2]. Good health and satisfying lives are determined both by “freedom from” want (basic subsistence and educational needs) and by “freedom to” undertake activities of one's choice to achieve personal goals [3]. Because freedom from want is dependent, at least to some extent, on the actions of others [4], achieving the goal of greater equity means that we must address the tension between individual freedom and solidarity. Liberty with responsibility exclusively to the self contradicts a view of social democracy that emphasizes that individuals arise from and are shaped by their societies, that their freedom to choose is embedded in social attachments, and that their social and economic rights must acknowledge solidarity as a balance between rights and responsibilities to themselves and others [5]. Solidarity is not discovered by reflection and reasoning, but rather by increasing our sensitivity (empathy) and adequate responses to the pain, suffering, and humiliation of others [6].

## Extending the Bioethics Discourse in a Globalized World

Until the 1960s, discussions about ethics were largely confined to philosophical and theological studies. Advances in technology and medicine, together with increased concern for individual rights and freedoms, led to a new bioethics in which theologians, philosophers, lawyers, and other scholars engaged in a public discourse on applied ethics. Initially, this favored biomedical issues at the level of individual health—for example death and dying, reproductive medicine, and research ethics.

However, since the birth of modern bioethics in the 1960s, the world has changed profoundly. Widening economic disparities, rapid population growth, the emergence of new infectious diseases, escalating ecological degradation, numerous local and regional wars, a stockpile of nuclear weapons, massive dislocations of people, advances in science and technology with profound implications for individuals and populations, and, most recently, new terrorist threats to life have demonstrated how interconnected we all are [7].

Growing global instability and threats from the widening gulf between the world's haves and have-nots call for new ways of thinking and acting. Distinctions between domestic and foreign policy have become blurred, and public health, even in the most privileged nations, is more closely linked than ever to health and disease in impoverished countries. The need for coherence between domestic and foreign policy was acknowledged by President Clinton when he declared HIV/AIDS a global emergency, and also by subsequent endeavors to foster a global response to this pandemic. Now, more than ever before, local action must be linked to a new global health ethics based on shared values to help make the world a more stable place. Such a new approach could facilitate transformation of current ideas about governance, the global political economy, and relations between countries.

A framework that combines an understanding of global interdependence with enlightened long-term self-interest has the potential to produce a broad spectrum of beneficial outcomes, especially in the area of global health. An extended public debate, promoted by building capacity for this process through a multidisciplinary approach to ethics in education and daily life, could be the driving force for such change. These changes require that interest in health and ethics be extended beyond the microlevel of interpersonal relationships and individual health to include ethical considerations regarding public/population health at the level of institutions, nations, and international relations.

Extending the discourse in this way could promote the new mindset needed to improve health and deal with threats to health on a global level. That mindset requires recognition that health, human rights, economic opportunities, good governance, peace, and development are all intimately linked within a complex, interdependent world. The challenge of the 21st century is to explore these links, to understand their implications, and to develop processes that can harness economic growth to human development, narrow global disparities in health, and promote peaceful coexistence.

## Five Transformational Approaches

A global agenda must thus extend beyond the rhetoric of universal human rights to include greater attention to duties, social justice, and interdependence. Health and ethics provide a framework within which such an agenda could be developed and promoted across borders and cultures. The relatively new interdisciplinary field of bioethics, when expanded in scope to embrace widely shared foundational values, could make a valuable contribution to the improvement of global health by providing the space for such a discussion to occur. Our vision, explicated in detail elsewhere, offers a way forward for global health reform through five transformational approaches [2].

### Developing a global state of mind

First, developing a global state of mind about the world and our place in it is perhaps the most crucial element in the development of an ethic for global health. Achieving this will require an understanding of the world as an unstable complex system [8,9], the balancing of individual goods and social goods, and the avoidance of harm to weak/poor nations through economic and other forms of exploitation that frustrate the achievement of human rights and well-being [10]. The emergence of a multi-faceted social movement, “globalization from below” (in which people at the grassroots around the world link up to impose their own needs and interests on the process of globalization), illustrates additional pathways to constructive change [11].

### Promoting long-term self-interest

Second, in arguing that it is both desirable and necessary to develop a global mindset in health ethics, we suggest that this change need not be based merely on altruism, and that promoting long-term self-interest is also essential if we acknowledge that lives across the world are inextricably interlinked by forces that powerfully shape health and well-being. As an example, consider the long-term self-interest and mutual interdependence in the face of emerging new infectious diseases and microbial antibiotic resistance [12].

### Striking a balance between optimism and pessimism

Third, striking a balance between optimism and pessimism about globalization, solidarity, and progress will require a platform for dialogue among stakeholders, and a space where people can share different views about globalization. A broader conception of bioethics offers a basis for such a space.

### Developing capacity

Fourth, our vision for promoting an ethic for global health also features the development of capacity and a commitment to a broader discourse on ethics propagated through centers regionally and globally networked in growing and supportive North–South partnerships [13].

### Achieving widespread access to public goods

Fifth, achieving widespread access to education, basic subsistence needs, and work requires collective action, including financing (to make sure they are produced), and good governance (to ensure their optimum distribution and use) [14]. Constructing new ways of achieving economic redistribution is the key to resolving many global problems.

## Conclusion

While it would seem that nothing has changed since Lester Pearson noted over 30 years ago that “there can be no peace, no security, nothing but ultimate disaster, when a few rich countries with a small minority of the world's people alone have access to the brave, and frightening, new world of technology, science, and of high material living standards, while the large majority live in deprivation and want, cut off from opportunities of full economic development; but with expectations and aspirations aroused far beyond the hope of realizing them” [15], there is now perhaps a faint glimmer of hope that such progress is possible [16,17].
